# Cloning and Characterization of the Mycovirus MpChrV2 from *Macrophomina phaseolina*

**DOI:** 10.3390/jof11090675

**Published:** 2025-09-12

**Authors:** Peimeng Sun, Luyang Song, Mengyuan Mu, Jiayi Ma, Xinyu Li, Kunni Tian, Mengyuan Zhang, Mingyue Zhang, Yuanyuan Zhang, Caiyi Wen, Jing Wang, Ying Zhao

**Affiliations:** 1 College of Plant Protection, Henan Agricultural University, Zhengzhou 450046, China; sunpeimeng2001@163.com (P.S.); lysong@henau.edu.cn (L.S.); mumengyuan71@163.com (M.M.); majiayi202412@163.com (J.M.); lxinyu202412@163.com (X.L.); 15893026401@163.com (K.T.); zmy020305@163.com (M.Z.); 18300765193@163.com (M.Z.); eryazhang5@163.com (Y.Z.); wencaiyi@henau.edu.cn (C.W.); 2 Key Laboratory of Integrated Pest Management on Crops in Southern Region of North China, Henan Key Laboratory of Agricultural Pest Monitoring and Control, Institute of Plant Protection, Henan Academy of Agricultural Sciences, Zhengzhou 450002, China

**Keywords:** *Macrophomina phaseolina*, *Chrysoviridae*, hypovirulence, biological control

## Abstract

*Macrophomina phaseolina* is a widely distributed soilborne phytopathogenic fungus that causes destructive diseases such as charcoal rot and stem canker, posing serious threats to crop yield and quality. In recent years, mycoviruses have gained attention as potential biological control agents. In this study, a novel double-stranded RNA (dsRNA) virus was identified from *M. phaseolina* strain 22C-8, isolated from sesame (*Sesamum indicum* L.) charcoal rot samples in Fuyang, Anhui Province, China. The viral genome comprised four dsRNA segments, each encoding a single open reading frame (ORF) predicted to encode RNA-dependent RNA polymerase (RdRp), coat protein (CP), and two hypothetical proteins. Phylogenetic analysis classified the virus as a new member of the genus *Betachrysovirus* in the family *Chrysoviridae*, and it was designated Macrophomina phaseolina chrysovirus 2 (MpChrV2). Pathogenicity assays in sesame seedlings revealed that MpChrV2 infection significantly reduced the virulence of *M. phaseolina* strain 22C-8. In contrast, virus-free derivatives (22C-8-VF18), obtained via protoplast regeneration, caused more severe symptoms and exhibited enhanced growth rates, indicating that MpChrV2 alters fungal physiology and pathogenicity. These findings suggest that MpChrV2 possesses a typical hypovirulence phenotype and holds promise as a biocontrol agent for sesame charcoal rot.

## 1. Introduction

Mycoviruses, obligate intracellular parasites that infect fungi, replicate and propagate within their fungal hosts [1]. These viruses are ubiquitously distributed across major fungal phyla, including Ascomycota, Basidiomycota, Chytridiomycota, Zygomycota, and Neocallimastigomycota. According to the International Committee on Taxonomy of Viruses (ICTV), mycoviral genomes are categorized into five types: single-stranded DNA (ssDNA), single-stranded RNA–reverse transcribing (ssRNA-RT), negative-sense single-stranded RNA (−ssRNA), positive-sense single-stranded RNA (+ssRNA), and double-stranded RNA (dsRNA) [2,3,4,5]. Among these, dsRNA viruses are further classified into seventeen families: *Alternaviridae, Amalgaviridae, Botybirnaviridae, Chrysoviridae, Curvulaviridae, Fusagraviridae, Megabirnaviridae, Megatotiviridae, Monocitiviridae, Orthototiviridae, Partitiviridae, Phlegiviridae, Polymycoviridae, Pseudototiviridae, Quadriviridae, Spinareoviridae,* and *Yadonushiviridae* [6]. Advancements in high-throughput sequencing and virome analysis have revolutionized mycovirus discovery, leading to the identification of numerous novel viruses and predicting the discovery of even more taxa in the future [7,8].

Sesame (*Sesamum indicum* L.), an ancient oilseed crop, is highly valued for its nutritional and medicinal properties. Rich in proteins, lipids, minerals, and vitamin E, sesame exhibits antioxidant, cholesterol-lowering, anti-inflammatory, hepatoprotective, nephroprotective, and antitumor activities. These attributes drive its extensive use in the food, pharmaceutical, and cosmetic industries. Additionally, sesame meal serves as animal feed and fertilizer, and sesame oil can be converted into biodiesel [9,10,11,12]. However, sesame cultivation faces significant threats from diseases, particularly charcoal rot caused by *M. phaseolina*. This disease manifests as basal stem browning and rot, leading to plant wilting, especially under hot and humid conditions. Other pathogens, such as *Alternaria alternata* causing fruit blight and *Xanthomonas euvesicatoria* pv. sesami causing leaf spot, further threaten sesame yields. Despite the different pathogens involved, integrated disease management strategies share common principles of prevention, early intervention, and multi-modal control [13,14]. Yet, the development of fungicide resistance calls for alternative, sustainable control methods.

*Sesamum indicum* L., an ancient oleaginous crop, remains a pivotal component of the global oilseed economy due to its high seed oil content (45–60%), rich profile of unsaturated fatty acids (e.g., linoleic acid), and bioactive lignan antioxidants (e.g., sesamin) [15,16]. However, its production is severely constrained by *M. phaseolina*-induced stem and root rot, which can cause yield losses of up to 80% in susceptible genotypes. Current management practices are predominantly reliant on synthetic fungicides (e.g., carbendazim, thiabendazole) applied via seed treatment or soil drenching. While effective at reducing initial disease incidence, prolonged use of these agents has led to the emergence of fungicide-resistant pathogen populations and unintended ecological consequences, including soil microbiome dysregulation and groundwater contamination [17,18].

*Macrophomina phaseolina*, a soilborne phytopathogenic fungus with a cosmopolitan distribution, infects over 500 plant species, including economically important crops like sesame (*Sesamum indicum* L.), soybean (*Glycine max*), and sunflower (*Helianthus annuus*). It causes devastating diseases such as charcoal rot and root rot [19,20]. The microsclerotia of *M. phasolina* could survive in soil for up to 15 years, endowing it with remarkable tolerance to high temperatures and drought, which allows it to thrive in diverse climates, from tropical to temperate regions [21]. In the primary stage, *M. phasolina* secretes cell wall-degrading enzymes and phytotoxins, which disrupt host tissues and suppress plant immune responses [22]. The taxonomic status of *M. phasolina* belongs to the family *Botryosphaeriaceae* and exhibits high genetic diversity, enabling broad host adaptation [23]. This pathogen has inflicted severe economic losses in agriculture. For example, peanut crops can suffer complete yield failure when infected at the pre-emergence stage [24]. Due to the resilience of microsclerotia, effective eradication remains a challenge. Current management strategies rely on cultural practices, chemical fungicides, and ecological regulation. Notably, despite extensive research, no mycoviruses have been exploited as biocontrol agents against *M. phaseolina* [20,25].

*M. phaseolina* is a cosmopolitan soilborne phytopathogen with remarkable ecological plasticity, characterized by hyaline, septate hyphae bearing thin, melanized cell walls ranging in color from pale tawny to dark fulvous [26,27]. As a polyphagous pathogen, it causes significant economic losses in warm–arid agroecosystems, affecting major crops such as *Glycine max*, *Gossypium* spp., and *Sesamum indicum* L. through the development of charcoal rot and basal stem rot. Its physiological tolerance to high temperatures, desiccation, and the formation of persistent microsclerotia in soil render conventional fungicide-based management strategies largely inefficient, exacerbating yield losses [28]. Virulence in *M. phaseolina* is multifactorial, governed by traits such as hyphal growth velocity, melanin biosynthesis capacity, and microsclerotial morphometry—strains exhibiting rapid vegetative growth and reduced microsclerotial diameter typically display enhanced pathogenicity [28]. Notably, despite extensive mycological investigations, a relatively small number of mycoviruses have been identified in this species, representing a critical knowledge gap in the study of viral modulation of fungal pathogenicity.

Mycoviruses have shown great promise as biocontrol agents due to their ability to induce hypovirulence in phytopathogenic fungi [2,29]. For example, Cryphonectria hypovirus 1 (CHV1) has been successfully used to manage chestnut blight [30], and Sclerotinia sclerotiorum hypovirulence-associated DNA virus 1 (SsHADV-1) controls stem rot in *Brassica napus*, with infected strains even promoting plant growth [31]. Within the family *Chrysoviridae*, members like Magnaporthe oryzae chrysovirus 1-A (MoCV1-A) and 1-B (MoCV1-B) significantly affect fungal pathogenicity, growth, and morphology [32]. The ORF4 protein of MoCV1-A, when heterologously expressed in *Saccharomyces cerevisiae,* causes cellular damage and inhibits *M. oryzae* spore germination, suggesting its potential as an antifungal agent [33]. However, the effects of chrysoviruses on fungal virulence vary; for instance, Alternaria alternata chrysovirus 1 (AaCV1) enhances virulence, while Pestalotiopsis theae chrysovirus 1 (PtCV1) renders the fungus nonpathogenic and boosts plant resistance [34]. Several mycoviruses have been identified in the genus *Macrophomina*, including MpFV1, MpEV1-2, and others [35,36]. Four chrysoviruses also have been discovered in *M. phaseolina*, but only the full-length sequence of the Macrophomina phaseolina chrysovirus 1 (MpChrV1) was cloned; for the other three viruses, only partial sequence information was obtained through high-throughput sequencing. Not only that, but none of them involved the analysis of hypovirulence characteristics [37,38].

In this study, we identified a novel mycovirus, Macrophomina phaseolina chrysovirus 2 (MpChrV2), from *M. phaseolina* infecting sesame in Fuyang, Anhui Province, China. Based on sequencing and phylogenetic analysis, we characterized MpChrV2 as a new member of the *Chrysoviridae* family. Our research aimed to confirm the hypovirulence-inducing effect of MpChrV2 on *M. phaseolina* and evaluate its potential as a biocontrol agent for sesame charcoal rot, offering a sustainable solution to this persistent agricultural challenge.

## 2. Materials and Methods

### 2.1. Strains and Culture Conditions

A total of five fungal strains (22C-8, G123, 22C-8VF-2, 22C-8VF-11, 22C-8VF-18) were utilized in this investigation. Strain 22C-8 was isolated from a *Sesamum indicum* L. plant exhibiting charcoal rot symptoms in Anhui Province, China. Strain G123 is a pathogenic strain of *Macrophomina phaseolina* that is associated with sesame stem blight. The virus-free derivatives 22C-8VF-2, 22C-8VF-11, and 22C-8-VF18 were generated from parental strain 22C-8 through protoplast regeneration. All strains were propagated on potato dextrose agar (PDA) medium under 28 °C in complete darkness for 72 h, and were subsequently stored in 20% (*v*/*v*) glycerol at −80 °C for long-term storage.

### 2.2. Extraction and Purification of dsRNA

The isolation and purification of double-stranded RNA (dsRNA) were conducted following the method of Zhao et al. [38]. Fresh mycelia were cultured for 3–4 days on PDA medium covered with sterile cellophane membranes to facilitate easy harvesting. Mycelia were collected and immediately snap-frozen in liquid nitrogen and ground into a fine powder using a mortar and pestle. DsRNA was specifically captured via affinity binding to cellulose powder C6288 (Sigma-Aldrich, Shanghai, China). To eliminate genomic DNA and single-stranded RNA contaminants, the extracted dsRNA was sequentially digested with RNase-free DNase I and S1 nuclease (Takara, Dalian, China). The purified dsRNA was then resolved by electrophoresis on a 1% agarose gel at 110 V for 2 h. Nucleic acid bands were visualized using SuperRed staining (Seven, Beijing, China) and documented with a gel imaging system (InGenius LhR, Syngene, Cambridge, UK).

### 2.3. cDNA Synthesis, Cloning, and Sequencing

Complementary DNA (cDNA) synthesis from purified double-stranded RNA (dsRNA) was carried out following the protocol described by Zhao et al. [38]. Briefly, first-strand cDNA was synthesized using the random primer RACE3RT and HiScript III reverse transcriptase (Vazyme, Nanjing, China). Subsequent PCR amplification was performed with the RACE3 primer. Amplified products were sequenced, and the resulting sequence data were used to design specific primers for RT-PCR. Sequence assembly was performed using SnapGene version 7.0.

To determine the terminal sequences of the dsRNA segments, the PC3-T7 loop primer was ligated to both the 5′ and 3′ ends of the dsRNA using T4 RNA ligase. RT-PCR was then conducted with segment-specific and PC2 primers. All amplified products were cloned into the pMD19-T vector and transformed into *Escherichia coli* DH5α for Sanger sequencing.

Sequencing was completed by Shanghai Bioengineering Co., Ltd. (Zhengzhou, China). Each nucleotide position of the full-length cDNA sequences was confirmed by at least three independent reads to ensure sequence accuracy. The primers used for cDNA amplification and sequencing are listed in Supplementary Table S1.

### 2.4. Sequence and Phylogenetic Analysis

Open reading frames (ORFs) and conserved protein domains within the dsRNA segments were predicted using the ORF Finder and Conserved Domain (CD) Search tools available on the NCBI website (https://www.ncbi.nlm.nih.gov). Multiple sequence alignments were conducted with representative viruses from related taxa using CLUSTALX version 2.0 [39]. Phylogenetic relationships were inferred using the maximum likelihood (ML) method with 1000 bootstrap replicates implemented in MEGA12. The phylogenetic trees were edited and graphically visualized using the Interactive Tree of Life (iTOL, https://itol.embl.de).

### 2.5. Virus-Free Fungal Strains Obtained via Protoplast Regeneration

In this study, a virus-free derivative, 22C-8-VF18, was generated from *Macrophomina phaseolina* strain 22C-8 via protoplast regeneration. The parental strain was cultured for 2 days on PDA plates covered with sterile cellophane, after which fresh mycelia were harvested and ground using a mortar. The resulting homogenate was inoculated into 100 mL of potato dextrose broth (PDB) and incubated overnight at 28 °C with agitation at 180 rpm. Protoplasts were isolated according to the protocol described by Nahalkova and Fatehi [39]. Following dilution, protoplasts were spread onto regeneration medium, and emerging hyphal tips were isolated to obtain protoplast-derived progeny of strain 22C-8. The successful curing of the mycovirus was validated by dsRNA extraction and reverse transcription PCR (RT-PCR) analysis.

### 2.6. RNA Extraction and RT-PCR Detection

To extract total RNA, *M. phaseolina* strains were cultured for 2–3 days on PDA plates covered with sterile cellophane. Fresh mycelia were collected and rapidly frozen in liquid nitrogen, followed by thorough grinding into a fine powder. Total RNA was extracted using an RNA isolation reagent (TransGen Biotech, Beijing, China) according to the manufacturer’s protocol.

For RT-PCR analysis, first-strand complementary DNA (cDNA) synthesis was carried out using the HiScript III 1st Strand cDNA Synthesis Kit with gDNA wiper (Vazyme Biotech Co., Ltd., Nanjing, China), following the supplier’s instructions. PCR amplification was conducted using virus-specific primers designed based on the MpChrV2 genomic sequence (listed in Supplementary Table S1) to detect virus-infected strains. Amplified products were resolved by electrophoresis on a 1.0% agarose gel and visualized accordingly.

### 2.7. Biological Properties of Fungal Isolates

#### 2.7.1. Mycelial Growth and Colony Morphology

To assess the effect of the mycovirus on its fungal host, we compared colony morphology, growth rate, and virulence between the virus-infected strain and its virus-free counterpart. A mycelial plug with a diameter of 0.5 cm was excised using a sterile cork borer and cultured onto 15 mL of PDA medium. Colony morphology was observed and photographed at one day post-inoculation. Colony diameter was measured every day to monitor growth rate.

#### 2.7.2. Pathogenicity Assay

Pathogenicity assays were conducted using potted sesame seeding. Three seedlings with the same growth status were selected for virulence testing. The apex of each seedling stem was excised approximately 20 mm above the cotyledonary node using a sterile razor blade. Each seedling served as a single replicate, with 15 replicates per *M. phaseolina* isolate. Mycelial plugs were harvested from the colony margin using a sterile 200 µL pipette tip and affixed directly to the apical stem wound, ensuring intimate contact between the plug and stem tissue. Seedlings were misted with sterile water three times each day (morning, noon, and evening) to maintain humidity. Pipette tips were removed after 48 h post-inoculation. For detached leaf assays, two mycelial plugs with a diameter of 0.5 cm were placed on both sides of the leaf vein using sterile pipette tips. Disease progression was subsequently documented through photographic records. The cut-stem inoculation method was performed with minor modifications [40].

To systematically evaluate the impact of MpChrV2 on the virulence of its host, a controlled pot experiment was designed using sesame (*Sesamum indicum* L.) as the model host plant. The experimental setup included three treatment groups: (1) seedlings and leaves inoculated with the MpChrV2-infected *M. phaseolina* strain 22C-8; (2) those inoculated with the virus-free *M. phaseolina* strain G123; and (3) an untreated control (CK) groups, allowing for a direct comparison of plant growth and disease development across different infection statuses.

## 3. Results

### 3.1. The Biological Characteristics of Strain 22c-8

**Figure 1:**
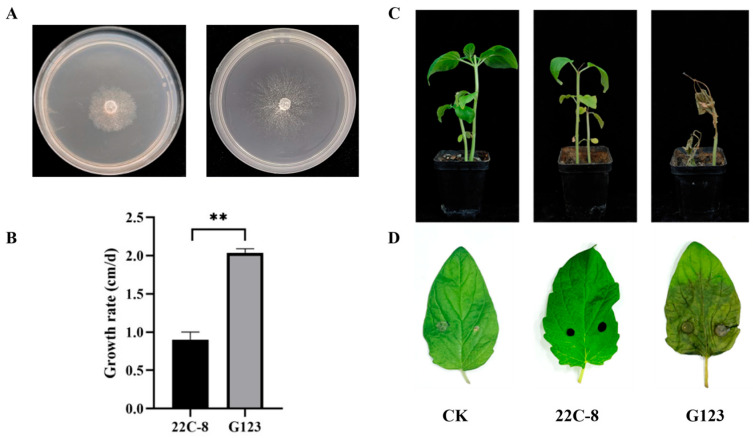
Characterization of strain 22C-8 and G123 and their biological effects on sesame. (**A**) Colony morphology of strain 22C-8 and G123. (**B**) Colony diameters of strain 22C-8 and G123. Significance was determined by paired *t*-test. ** indicate highly significant differences (*p* < 0.01). (**C**) Sesame plants were inoculated with 22C-8 and G123 strains, with untreated plants as controls, and their growth status was observed 10 days post-inoculation. (**D**) Sesame leaves were inoculated with 22C-8 and G123 strains, with untreated plants as controls, and their growth status was observed 4 days post-inoculation.

During strain isolation procedures, comparative growth analysis revealed that strain 22C-8 displayed an aberrant growth rate relative to the characterized wild-type strain G123 (Figure 1A). The colony morphology and radial growth rate of *Macrophomina phaseolina* strain 22C-8 and the wild-type strain G123 were compared on PDA medium. After 24 h of incubation, strain 22C-8 exhibited a colony expansion rate of 0.90 cm/d, whereas strain G123 displayed a significantly higher growth rate of 3.38 cm/d, and the growth rate of strain 22C-8 decreased compared with that of strain G123 (Figure 1B). Consequently, comparative pathogenicity assays were conducted between strain 22C-8 and the reference wild-type strain G123.

Six days following seedling inoculation, the control groups exhibited normal growth characteristics, with vibrant green leaves and no visible signs of disease. In contrast, plants in the 22C-8 treatment group showed stunted growth characterized by mild chlorosis. Despite the compromised foliage, these plants retained an upright posture, indicating a relatively moderate impact on their structural integrity. Conversely, the G123 treatment group suffered severe damage, with leaves turning yellow, wilting significantly, and a high mortality rate of 44% among the seedlings, visually evident in Figure 1C.

When assessing the leaf inoculation outcomes four days post-inoculation, the CK leaves remained healthy and green without any lesions. In the 22C-8 treatment, only the fungal inoculation plugs were visible on the leaf surfaces, with no discernible disease spots developing. This suggests that the presence of MpChrV2 may inhibit the pathogenicity of *M. phaseolina* strain 22C-8. In stark contrast, the G123-inoculated leaves displayed extensive damage, including yellowing, blackening, and the formation of large necrotic lesions, presenting the most severe disease phenotypes (Figure 1D).

Collectively, analysis of seedling and foliar inoculation assays suggested the potential presence of mycoviral infection in strain 22C-8. Therefore, to confirm the presence of mycoviruses, the extraction of dsRNAs was carried out.

### 3.2. Double-Stranded RNA Segments in Strain 22C-8

**Figure 2:**
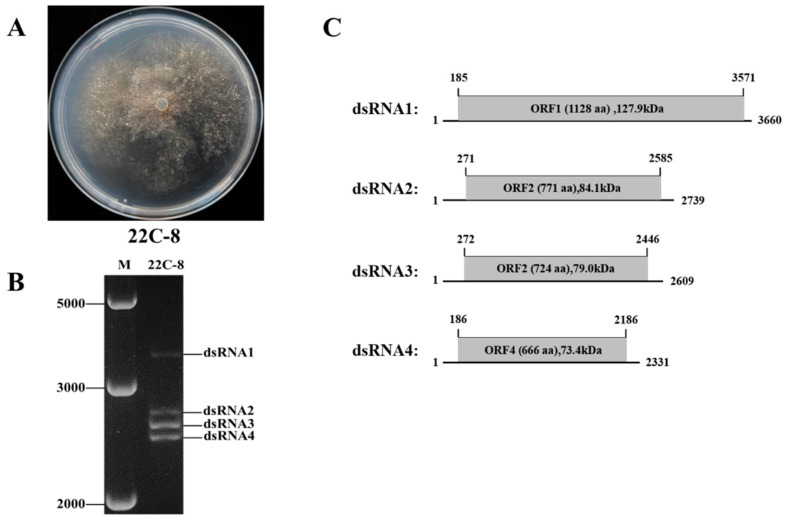
(**A**) Colony morphology of the *Macrophomina phaseolina* strain 22C-8. (**B**) Agarose gel electrophoresis of dsRNA treated with DNase I and S1 nuclease. (**C**) Genome diagram of MpChrV2.

Strain 22C-8 of *M. phaseolina*, a single-conidium isolate derived from the hypovirulent strain 2012–22 [36], contained detectable dsRNA (MpChrV2) and it was confirmed to a hypovirulent strain (Figure 2A). Following enzymatic treatment with DNase I and S1 nuclease to eliminate potential DNA and single-stranded RNA contaminants, four dsRNA were detected by agarose gel (1%) (Figure 2B). The sizes of these dsRNA segments were determined to be approximately 3.7 kb, 2.7 kb, 2.6 kb, and 2.3 kb, respectively (Figure 2C).

### 3.3. Molecular Characterization of MpChrV2 in Strain 22C-8

The four dsRNA segments of strain 22C-8 have been cloned, sequenced, and deposited in the NCBI GenBank database with accession numbers MT035905, PX239992, PX239993, and MT035909.

The full-length cDNA of dsRNA1 was 3660 bp in length with a GC content of 49%. The dsRNA1 contained one large ORF1 (185–3571 nt) encoding an RdRp of 1128 aa. The 5′-UTR (untranslated regions) and 3′-UTR of dsRNA1 were 184 nt and 89 nt, respectively. The molecular weight of the predicted RdRp was 127.9 kDa, which contained the RdRp_4 (pfam02123, 323–832 aa) domain. A BLASTp analysis indicated that this protein shared the highest similarity with the RdRp of Aspergillus mycovirus 1816 (ABX79996.1), exhibiting an identity of 48.86% (E-value = 0) (Table 1).

The full-length cDNA of dsRNA2 was 2738 bp in length with a GC content of 53%. The dsRNA2 contained one large ORF2 (271–2585 nt) encoding a CP of 771 aa. The 5′-UTR and 3′-UTR were 270 nt and 153 nt, respectively. The molecular weight of predicted CP was 84.1 kDa, which contained a CP (PRK10811, 548–680 aa) domain. A BLASTp analysis showed that this protein shared the highest similarity with the coat protein encoded by Erysiphe necator-associated chrysovirus 1 (QKK35385.1), exhibiting an identity of 49.35% and an (E-value = 0) (Table 1).

The full-length cDNA of dsRNA3 was 2609 bp in length with a GC content of 53%. The dsRNA3 contained one large ORF3 (272–2446 nt) encoding an HP (hypothetical protein) of 724 aa. The 5′-UTR and 3′-UTR were 271 nt and 163 nt, respectively. The molecular weight of predicted HP was 79.0 kDa. A BLASTp analysis revealed that this protein shared the highest similarity with the hypothetical protein encoded by Coniothyrium diplodiella chrysovirus 1 (YP_010088048.1), exhibiting an identity of 50.52% and an (E-value = 0) (Table 1).

The full-length cDNA of dsRNA4 was 2331 bp in length with a GC content of 51%. The dsRNA4 contained one large ORF4 (186–2186 nt) encoding an HP (hypothetical protein) of 666 aa. The 5′-UTR and 3′-UTR were 185 nt and 145 nt, respectively. The molecular weight of predicted HP was 73.4 kDa. A BLASTp analysis indicated that this protein shared the highest similarity with the hypothetical protein encoded by Setosphaeria turcica chrysovirus 1 (WAO97412.1), exhibiting an identity of 34.3% and an (E-value = 1.00 × 10^−117^) (Table 1). We predicted the tertiary structures of all four ORFs encoded by MpChrV2 using AlphaFold3, as shown in Figure S1.

**Figure 3:**
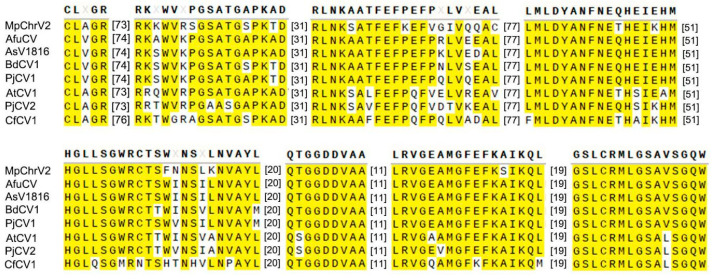
Multiple alignment of the conserved RdRp amino acid sequences encoded by MpChrV2 and other *Chrysoviridae* family members. The full names of these virus abbreviations are as follows: AfuCV, A. fumigatus chrysovirus; AsV1816, Aspergillus mycovirus 1816; BdCV1, Botryosphaeria dothidea chrysovirus 1; PjCV1, Penicillium janczewskii chrysovirus 1; AtCV-1, Acanthocystis turfacea chlorella virus 1; PjCV2, Penicillium janczewskii chrysovirus 2; CfCV1, Colletotrichum fructicola chrysovirus 1.

The RdRp sequence of the MpChrV2 isolated from *M. phaseolina* strain 22C-8 was comprehensively analyzed by performing conserved domain database searches and conducting multiple protein sequence alignments with other members in the family *Chrysoviridae*. As illustrated in Figure 3, the RdRp of the MpChrV2 contained eight conserved motifs and showed a high degree of conservation with those established representatives in the family *Chrysoviridae* [41].

### 3.4. Sequence Analysis of the 5′- and 3′-Untranslated Regions

**Figure 4:**
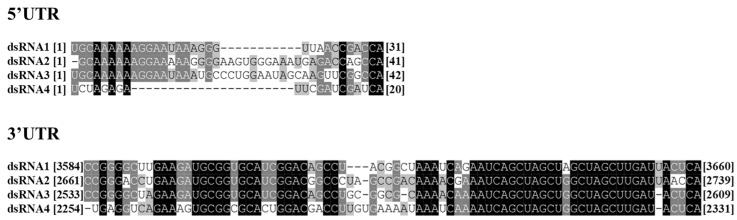
Multiple sequence alignment of MpChrV2 dsRNA segments in the 5′- and 3′-UTRs. Residue coloring indicates conservation levels: black for 100% identity, dark gray for 80% identity, light gray for 60% identity, and white for non-conserved positions.

The 5′-UTRs of coding strands of dsRNA 1–4 were 184, 270, 271, and 185 nt in length, respectively (Figure 1C). Comparative analysis of the 5′-UTR nucleotide sequences across all four dsRNA segments revealed significant sequence conservation. Specifically, 2 nt (CA) was completely conserved, while 12 nt (GCAAAAAAGGAA) exhibited strict conservation in dsRNA 1–3 of the MpChrV2 isolate (Figure 4). The evolutionary persistence of conserved UTR domains across viruses suggests a probable essential role for these regions in the viral replication cycle. The 3′-UTRs of coding strands of dsRNA 1–4 were 89, 153, 163, and 145 nt in length, respectively (Figure 1C). High sequence similarity was detected based on multiple nucleotide sequence alignment analyses of the 3′-UTR of the four dsRNA segments. The 3′-UTR regions contained two universally conserved elements, 12 nt (AAUCAGCUAGCU) and 11 nt (GCUAGCUUGAU), of dsRNA 1–4. Furthermore, dsRNA 1–3 exhibited strict conservation of 5 nt (CCGGG) and 20 nt (GAAGAUGCGGUGCAUCGGAC) (Figure 4).

### 3.5. Phylogenetic Analysis of MpChrV2

To precisely determine the taxonomic classification and elucidate the phylogenetic relationship of MpChrV2, a comprehensive comparative analysis was conducted. Specifically, the RdRp (ORF1) and CP (ORF2) protein sequences of MpChrV2 were aligned with those of representative members from the *Chrysoviridae* family. Additionally, protein sequences from selected members of the *Totiviridae* family were incorporated into the analysis and served as an outgroup, providing a crucial reference for inferring evolutionary divergence.

**Figure 5:**
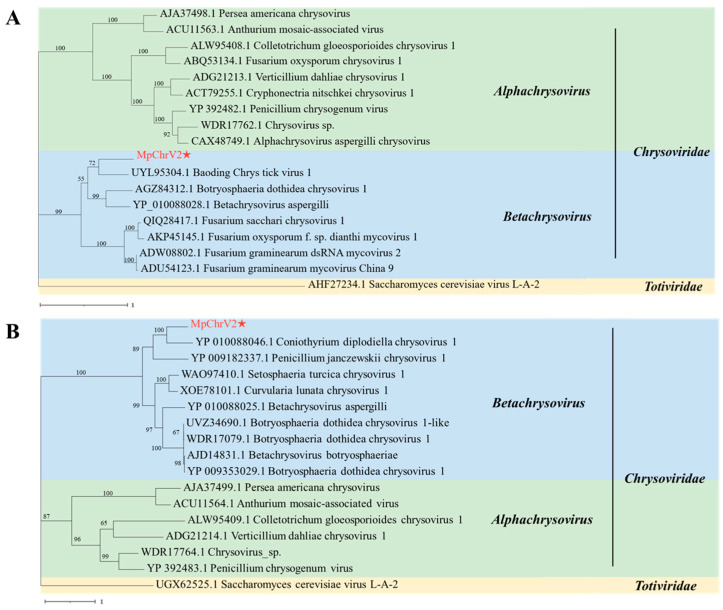
Phylogenetic analysis of MpChrV2 with other related viruses. (**A**) Phylogenetic analysis of the RdRp of MpChrV2 and other mycoviruses in the family *Chrysoviridae*. (**B**) Phylogenetic analysis of the CP of MpChrV2 and other mycoviruses in the family *Chrysoviridae*. The target virus is marked with “★”.

Maximum likelihood (ML) phylogenetic analysis using the JTT model was performed to reveal the evolutionary relationships among the viral isolates. The RdRp (ORF1) protein sequence of MpChrV2 exhibited a close evolutionary relationship with Baoding Chrys tick virus 1 (exhibiting an identity of 47.63%), forming a distinct clade. MpChrV2 further clustered with Botryosphaeria dothidea chrysovirus 1 and Betachrysovirus aspergilli, and these viruses, in turn, grouped together with Fusarium sacchari chrysovirus 1, Fusarium oxysporum f. sp. dianthi mycovirus 1, Fusarium graminearum dsRNA mycovirus 2, and Fusarium graminearum mycovirus China 9, forming a well-supported major clade within the genus *Betachrysovirus* of the family *Chrysoviridae* (Figure 5A). Similarly, the CP (ORF2) protein sequence of MpChrV2 showed the closest evolutionary affinity with that of Coniothyrium diplodiella chrysovirus 1 (exhibiting an identity of 45.05%). These two viruses formed a tight branch that was most closely related to Penicillium janczewskii chrysovirus 1, with which they assembled into a small monophyletic group. This group further clustered with other Betachrysovirus members such as Setosphaeria turcica chrysovirus 1, Curvularia lunata chrysovirus 1, and Betachrysovirus aspergilli, forming a larger evolutionary branch. This branch was further related to additional viruses including Botryosphaeria dothidea chrysovirus 1-like, Botryosphaeria dothidea chrysovirus 1, and Betachrysovirus botryosphaeriae, all of which are classified within the *Betachrysovirus* genus of the family *Chrysoviridae* (Figure 5B). We also performed phylogenetic analysis on the proteins encoded by ORF3 and ORF4, as shown in Figure S2.

The phylogenetic analysis results clearly demonstrate that MpChrV2 clusters distinctly with members of the *Betachrysovirus* genus. This strong phylogenetic signal, combined with the high bootstrap support values at key nodes, provides compelling evidence that MpChrV2 represents a novel member of the *Betachrysovirus* genus within the *Chrysoviridae* family, thereby expanding the known diversity of this viral taxonomic group.

### 3.6. Derivation of Virus-Free Fungal Strains

In order to explore the hypovirulent mechanism of MpChrV2 on its fungal host, additional experiments were carried out to eliminate MpChrV2 from *M. phaseolina* strain 22C-8 through protoplast regeneration techniques. A total of ten regenerated strains were successfully obtained, and subsequent reverse transcription polymerase chain reaction (RT-PCR) analysis confirmed that five of these strains were free of MpChrV2.

**Figure 6:**
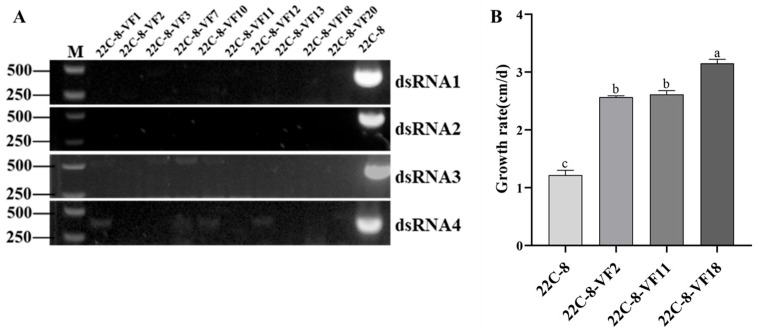
(**A**) The RT-PCR of strain 22C-8 and its derivative strains. (**B**) Comparison the growth rate of strain 22C-8 and its derivative strains. Statistical analysis was performed using one-way analysis of variance (ANOVA). Different lowercase letters indicate statistically significant differences (Tukey’s post hoc test, *p* < 0.001), while shared letters indicate no significant difference.

Three virus-free strains (22C-8-VF2, 22C-8-VF11, and 22C-8-VF18) were selected for radial growth rate assessment. The measured growth rates were 2.57 cm/d for 22C-8-VF2, 2.62 cm/d for 22C-8-VF11, and 3.15 cm/d for 22C-8-VF18, all significantly exceeding the 1.22 cm/d observed in the parental strain 22C-8 (Figure 6). Among them, strain 22C-8-VF18 was selected for further investigation.

### 3.7. Biological Effects of MpChrV2 on Macrophomina phaseolina Strain 22C-8

**Figure 7:**
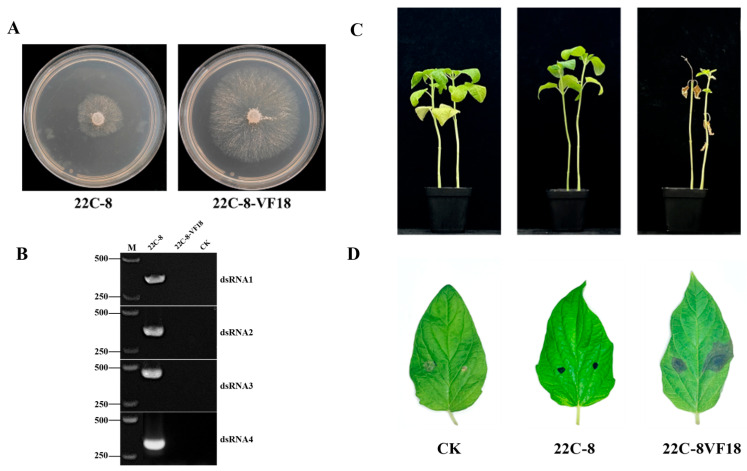
Characterization of MpChrV2-infected strain 22C-8 and cured strain 22C-8-VF18 of *M. phaseolina*. (**A**) Colony morphology of strain 22C-8 and 22C-8-VF18. (**B**) RT-PCR of strain 22C-8 and 22C-8-VF18. (**C**) Sesame plants were inoculated with strain 22C-8 and 22C-8-VF18, with untreated plants as control, and their growth status was observed 10 days post-inoculation. (**D**) Sesame leaves were inoculated with stain 22C-8 and 22C-8-VF18, with untreated plants as control, and their growth status was observed after 4 days post-inoculation.

After a 24 h incubation period on PDA medium, the colony morphology of both the parental wild-type strain 22C-8 and the virus-free strain 22C-8-VF18 were examined (Figure 7A,B).

To further validate the hypothesis that MpChrV2 infection reduces the virulence of *M. phaseolina*, a series of inoculation experiments were conducted on sesame seedlings and leaves. The experimental design included inoculation with the 22C-8 (MpChrV2-infected) strain and the 22C-8-VF18 (virus-free) strain, with untreated plants serving as the control groups.

Ten days post-seedling inoculation, the control (CK) groups displayed normal growth, characterized by lush green leaves and no visible signs of disease. In contrast, plants inoculated with the 22C-8 strain showed mild growth retardation, with a reduced number of leaves but no significant wilting. In contrast, the 22C-8-VF18-inoculated plants exhibited severe developmental defects, including wilting leaves and a notable mortality rate of 68%, as illustrated in Figure 7C.

Similarly, leaves remained healthy and green after 4 dpi in CK groups, whereas the 22C-8-inoculated leaves showed no disease symptoms, maintaining uniform coloration. Conversely, the 22C-8-VF18-inoculated leaves developed distinct lesions, characterized by the appearance of brown necrotic spots, as shown in Figure 7D.

Comparative phenomics with its virus-cured derivative (22C-8-VF18) revealed two distinct hypovirulence-associated phenotypes: (1) a statistically significant 23.7% reduction in hyphal extension rate, suggesting perturbation of energy metabolism or cell wall biogenesis pathways; and (2) substantial virulence attenuation on *Sesamum indicum* L., manifested as a 68% reduction in seedling mortality and 59% decrease in lesion area compared to the virus-free control (Figure 7C,D) [28].

Collectively, these results from both colony growth and plant inoculation assays provide compelling evidence that MpChrV2 infection significantly attenuates the virulence of *M. phaseolina*, effectively reducing the damage caused by sesame stem blight. This discovery underscores the crucial role of MpChrV2 in modulating host virulence and opens up new avenues for exploring this mycovirus as a promising biological control agent for managing *M. phaseolina*-associated plant diseases in agricultural settings.

## 4. Discussion

In recent years, the rapid advancement of high-throughput sequencing technologies has facilitated the discovery of numerous mycoviruses in phytopathogenic fungi, with an increasing body of research documenting their capacity to induce hypovirulence in fungal hosts [34,42]. These findings have not only expanded the mechanistic understanding of virus–fungal interactions but also stimulated interest in leveraging mycoviruses as biocontrol agents. Prior to this study, however, few mycoviruses had been documented in *M. phaseolina*—a soilborne ascomycete with an exceptionally broad host range (exceeding 500 plant species), responsible for devastating diseases such as charcoal rot. Here, we describe the first isolation, molecular characterization, and taxonomic assignment of a novel mycovirus from *M. phaseolina* strain 22C-8, designated Macrophomina phaseolina chrysovirus 2 (MpChrV2). In accordance with the latest taxonomic revisions by the International Committee on Taxonomy of Viruses (ICTV), the family *Chrysoviridae* comprises two genera: *Alphachrysovirus* and *Betachrysovirus* [43,44]. Through our analysis of genome architecture annotation, conserved domain profiling, and phylogenetic inference, we robustly placed MpChrV2 within the *Betachrysovirus* genus. This study represents a report of a chrysovirus infecting *M. phaseolina*.

This study addresses this gap through the first isolation and functional characterization of MpChrV2, a novel *Chrysoviridae* member, from strain 22C-8. Collectively, these observations suggest that MpChrV2 exerts its hypovirulent effects by interfering with conserved virulence determinants in *M. phaseolina*. The discovery of MpChrV2 not only broadens the host range of the *Chrysoviridae* but also provides a rare example of mycovirus-mediated hypovirulence in a soilborne fungus lacking known vertical transmission mechanisms. Given the significant agronomic and ecological impacts of *M. phaseolina*, these findings underscore the biocontrol potential of MpChrV2, particularly in sustainable agriculture systems prioritizing reduced chemical input.

Mycovirus-mediated hypovirulence has emerged as a transformative strategy in plant disease management, with well-characterized examples including Cryphonectria hypovirus 1 (CHV1) for chestnut blight control and Sclerotinia sclerotiorum hypovirulence-associated DNA virus 1 (SsHADV1) for white mold management [21,45]. Despite the economic and nutritional importance of oilseed crops like sesame, mycovirus research in these systems remains underexplored. The identification of MpChrV2 addresses this disparity, demonstrating a novel mycovirus that not only reduces *M. phaseolina* virulence but also reprograms host physiology, as evidenced by altered colony pigmentation and growth dynamics. These phenotypic alterations are hypothesized to arise from viral interference with conserved virulence pathways, such as melanin biosynthesis (critical for microsclerotia viability) and mitochondrial energy metabolism (essential for hyphal tropism).

From a translational perspective, the hypovirulent phenotype of MpChrV2 offers a science-based alternative to conventional chemical control, particularly in organic and low-input farming systems. Future research priorities should include the following: (1) elucidating the mechanisms of horizontal and vertical viral transmission in soil–plant systems; (2) identifying viral effector proteins involved in virulence attenuation through yeast two-hybrid screening and comparative proteomics; and (3) assessing the ecological safety and field stability of MpChrV2 in multispecies fungal communities. Addressing these objectives will not only advance fundamental knowledge of virus–fungal coevolution but also facilitate the development of MpChrV2 as a next-generation biocontrol agent for *M. phaseolina*-related diseases in sesame and other economically important crops.

## 5. Conclusions

In this study, a novel chrysovirus was identified from the *M. phaseolina* strain 22C-8 and designated Macrophomina phaseolina chrysovirus 2 (MpChrV2). Based on BLASTp analysis, multiple sequence alignment, and phylogenetic inference, MpChrV2 was classified as a new member of the genus *Betachrysovirus* within the family *Chrysoviridae*. This represents the first report of a chrysovirus associated with *M. phaseolina.* The MpChrV2 infection can cause hypovirulence to *M. phaseolina*, and it is also a potential biocontrol agent that could be further studied for controlling the root rot of *Sesamum indicum*.

## Figures and Tables

**Table 1:** Identity between MpChrV2-encoded proteins and equivalent proteins of other chrysoviruses.

MpChrV2	Predicted Protein	Best Match	Identity (%)	E-Value	Query Cover
dSRNA1	RdRp	Aspergillus mycovirus 1816	48.86	0	95%
		Aspergillus thermomutatus chrysovirus 1	48.49	0	99%
		Aspergillus fumigatus chrysovirus	48.2	0	97%
dSRNA2	CP	Erysiphe necator associated chrysovirus 1	49.35	0	99%
		Coniothyrium diplodiella chrysovirus 1	45.17	0	99%
		Penicillium janczewskii chrysovirus 1	36.05	5.00 × 10^−139^	94%
dSRNA3	HP	Coniothyrium diplodiella chrysovirus 1	50.52	0	79%
		Erysiphe necator associated chrysovirus 1	49.26	0	92%
		Neofusicoccum parvum chrysovirus 2	40	1.00 × 10^−117^	79%
dSRNA4	HP	Setosphaeria turcica chrysovirus 1	34.3	9.00 × 10^−51^	69%
		Bipolaris maydis chrysovirus 2	32.99	7.00 × 10^−53^	79%
		Penicillium janczewskii chrysovirus 1	32.49	1.00 × 10^−68^	58%

## Data Availability

Data are contained within the article and Supplementary Materials.

## Supplementary Materials

The following supporting information can be downloaded at: https://www.mdpi.com/article/10.3390/jof11090675/s1, Supplementary Figure S1: Predicted Tertiary Structure of MpChrV2; Supplementary Figure S2: Phylogenetic analysis of MpChrV2 with other related viruses; Supplementary Table S1: A list of primers used in this study.

## References

[1] Xie J., Jiang D. (2024). Understanding the Diversity, Evolution, Ecology, and Applications of Mycoviruses. Annu. Rev. Microbiol..

[2] Xie J., Jiang D. (2014). New insights into mycoviruses and exploration for the biological control of crop fungal diseases. Annu. Rev. Phytopathol..

[3] Ghabrial S.A., Castón J.R., Jiang D., Nibert M.L., Suzuki N. (2015). 50-plus years of fungal viruses. Virology.

[4] Kondo H., Botella L., Suzuki N. (2022). Mycovirus diversity and evolution revealed/inferred from recent studies. Annu. Rev. Phytopathol..

[5] Son M., Yu J., Kim K.H. (2015). Five Questions about Mycoviruses. PLoS Pathog..

[6] Jiang Y., Tian X., Liu X., Yang B., Wang N., Wang Q., Yu W., Qi X., Peng J., Hsiang T. (2022). Complete genome sequence of a novel chrysovirus infecting Talaromyces neofusisporus. Arch. Virol..

[7] Wang Q., Cheng S., Xiao X., Cheng J., Fu Y., Chen T., Jiang D., Xie J. (2019). Discovery of Two Mycoviruses by High-Throughput Sequencing and Assembly of Mycovirus-Derived Small Silencing RNAs From a Hypovirulent Strain of Sclerotinia sclerotiorum. Front. Microbiol..

[8] Sato Y., Suzuki N. (2023). Continued mycovirus discovery expanding our understanding of virus lifestyles, symptom expression, and host defense. Curr. Opin. Microbiol..

[9] Myint D., Gilani S.A., Kawase M., Watanabe K.N. (2020). Sustainable sesame (*Sesamum indicum* L.) production through improved technology: An overview of production, challenges and opportunities in Myanmar. Sustainability.

[10] Wei P., Zhao F., Wang Z., Wang Q., Chai X., Hou G., Meng Q. (2022). Sesame (*Sesamum indicum* L.): A Comprehensive Review of Nutritional Value, Phytochemical Composition, Health Benefits, Development of Food, and Industrial Applications. Nutrients.

[11] Mili A., Das S., Nandakumar K., Lobo R. (2021). A comprehensive review on *Sesamum indicum* L.: Botanical, ethnopharmacological, phytochemical, and pharmacological aspects. J. Ethnopharmacol..

[12] Takahashi M., Nishizaki Y., Sugimoto N., Takeuchi H., Nakagawa K., Akiyama H., Sato K., Inoue K. (2016). Determination and purification of sesamin and sesamolin in sesame seed oil unsaponified matter using reversed-phase liquid chromatography coupled with photodiode array and tandem mass spectrometry and high-speed countercurrent chromatography. J. Sep. Sci..

[13] Cheng H., Gao X., Sun H., Na Y., Xu J. (2021). First report of fruit blight caused by *Alternaria alternata* on sesame in Northeast China. Plant Dis..

[14] Noh E., Panth M., Yang X., Barnes J.M., Wang H. (2024). First Report of *Xanthomonas euvesicatoria* pv. *Sesame* Causing Leaf Spot on Sesame (*Sesamum indicum* L.) in South Carolina, USA. Plant Dis..

[15] Zech-Matterne V., Tengberg M., Van Andringa W. (2015). *Sesamum indicum* L. (Sesame) in 2nd Century BC Pompeii, Southwest Italy, and a Review of Early Sesame Finds in Asia and Europe. Veg. Hist. Archaeobotany.

[16] Yadav R., Kalia S., Rangan P., Pradheep K., Rao G.P., Kaur V., Pandey R., Rai V., Vasimalla C.C., Langyan S. (2022). Current Research Trends and Prospects for Yield and Quality Improvement in Sesame, an Important Oilseed Crop. Front. Plant Sci..

[17] Vyas S.C. (1981). Diseases in sesamum in India and their control. Pesticides.

[18] Shumaila S., Khan M.R. (2016). Management of root rot of mungbean caused by Macrophomina phaseolina through seed treatment with fungicides. Indian Phytopathol..

[19] Kaur S., Dhillon G.S., Brar S.K., Vallad G.E., Chand R., Chauhan V.B. (2012). Emerging phytopathogen Macrophomina phaseolina: Biology, economic importance and current diagnostic trends. Crit. Rev. Microbiol..

[20] Marquez N., Giachero M.L., Declerck S., Ducasse D.A. (2021). Macrophomina phaseolina: General characteristics of pathogenicity and methods of control. Front. Plant Sci..

[21] Shirai M., Eulgem T. (2023). Molecular interactions between the soilborne pathogenic fungus Macrophomina phaseolina and its host plants. Front. Plant Sci..

[22] Islam M., Haque M., Islam M., Emdad E., Halim A., Hossen Q.M., Hossain M.Z., Ahmed B., Rahim S., Rahman M.S. (2012). Tools to kill: Genome of one of the most destructive plant pathogenic fungi *Macrophomina phaseolina*. BMC Genom..

[23] Crous P.W., Slippers B., Wingfield M.J., Rheeder J., Marasas W.F.O., Philips A.J.L., Alves A., Burgess T., Barber P., Groenewald J.Z. (2006). Phylogenetic lineages in the Botryosphaeriaceae. Stud. Mycol..

[24] Sharma R.C., Bhowmik T.P. (1986). Estimation of yield losses in groundnut due to *Macrophomina phaseolina* (Tassi) Goid. Indian J. Plant Pathol..

[25] Gupta G.K., Sharma S.K., Ramteke R. (2012). Biology, epidemiology and management of the pathogenic fungus *Macrophomina phaseolina* (Tassi) goid with special reference to charcoal rot of soybean (*Glycine max* (L.) Merrill). J. Phytopathol..

[26] Cohen R., Omari N., Porat A., Edelstein M. (2012). Management of Macrophomina wilt in melons using grafting or fungicide soil application: Pathological, horticultural and economical aspects. Crop Prot..

[27] Lakhran L., Ahir R.R., Choudhary M., Choudhary S. (2018). Isolation, purification, identification and pathogenicity of Macrophomina phaseolina (Tassi) goid caused dry root rot of chickpea. J. Pharmacogn. Phytochem..

[28] Tok F.M. (2019). Relationship between Morphologic, Phenotypic and Pathogenic Characteristics in Macrophomina phaselina Isolates from Cucumber Plants. Int. J. Innov. Approaches Agric. Res..

[29] García-Pedrajas M.D., Cañizares M.C., Sarmiento-Villamil J.L., Jacquat A.G., Dambolena J.S. (2019). Mycoviruses in Biological Control: From Basic Research to Field Implementation. Phytopathology.

[30] Milgroom M.G., Cortesi P. (2004). Biological control of chestnut blight with hypovirulence: A critical analysis. Annu. Rev. Phytopathol..

[31] Urayama S., Kimura Y., Katoh Y., Ohta T., Onozuka N., Fukuhara T., Arie T., Teraoka T., Komatsu K., Moriyama H. (2016). Suppressive effects of mycoviral proteins encoded by Magnaporthe oryzae chrysovirus 1 strain A on conidial germination of the rice blast fungus. Virus Res..

[32] Okada R., Ichinose S., Takeshita K., Urayama S.I., Fukuhara T., Komatsu K., Arie T., Ishihara A., Egusa M., Kodama M. (2018). Molecular characterization of a novel mycovirus in Alternaria alternata manifesting two-sided effects: Down-regulation of host growth and up-regulation of host plant pathogenicity. Virology.

[33] Zhou L., Li X., Kotta-Loizou I., Dong K., Li S., Ni D., Hong N., Wang G., Xu W. (2021). A mycovirus modulates the endophytic and pathogenic traits of a plant associated fungus. ISME J..

[34] Chiba S., Kondo H., Kanematsu S., Suzuki N. (2010). Mycoviruses and virocontrol. Uirusu.

[35] Wang J., Xiao Y., Zhao H., Ni Y., Liu X., Zhao X., Wang G., Xiao X., Liu H. (2019). A novel double-stranded RNA mycovirus that infects Macrophomina phaseolina. Arch. Virol..

[36] Wang J., Ni Y., Liu X., Zhao H., Xiao Y., Xiao X., Li S., Liu H. (2020). Divergent RNA viruses in *Macrophomina phaseolina* exhibit potential as virocontrol agents. Virus Evol..

[37] Marzano S.L., Nelson B.D., Ajayi-Oyetunde O., Bradley C.A., Hughes T.J., Hartman G.L., Eastburn D.M., Domier L.L. (2016). Identification of Diverse Mycoviruses through Metatranscriptomics Characterization of the Viromes of Five Major Fungal Plant Pathogens. J. Virol..

[38] Zhao Y., Zhang Y., Wan X., She Y., Li M., Xi H., Xie J., Wen C. (2020). A Novel Ourmia-Like Mycovirus Confers Hypovirulence-Associated Traits on *Fusarium oxysporum*. Front. Microbiol..

[39] Nahalkova J., Fatehi J. (2003). Red fluorescent protein (DsRed2) as a novel reporter in Fusarium oxysporum f. sp. lycopersici. FEMS Microbiol. Lett..

[40] Twizeyimana M., Hill C.B., Pawlowski M., Paul C., Hartman G.L. (2012). A Cut-Stem Inoculation Technique to Evaluate Soybean for Resistance to Macrophomina phaseolina. Plant Dis..

[41] Zhai L., Zhang M., Hong N., Xiao F., Fu M., Xiang J., Wang G. (2018). Identification and Characterization of a Novel Hepta-Segmented dsRNA Virus From the Phytopathogenic Fungus Colletotrichum fructicola. Front. Microbiol..

[42] Umer M., Mubeen M., Shakeel Q., Ali S., Iftikhar Y., Bajwa R.T., Anwar N., Rao M.J., He Y. (2023). Mycoviruses: Antagonistic Potential, Fungal Pathogenesis, and Their Interaction with *Rhizoctonia solani*. Microorganisms.

[43] Zou C., Cao X., Zhou Q., Yao Z. (2024). The Interaction between Hypovirulence-Associated Chrysoviruses and Their Host *Fusarium* Species. Viruses.

[44] Kotta-Loizou I., Castón J.R., Coutts R.H.A., Hillman B.I., Jiang D., Kim D.H., Moriyama H., Suzuki N., Ictv Report Consortium (2020). ICTV Virus Taxonomy Profile: *Chrysoviridae*. J. Gen. Virol..

[45] Yu X., Li B., Fu Y., Xie J., Cheng J., Ghabrial S.A., Li G., Yi X., Jiang D. (2013). Extracellular transmission of a DNA mycovirus and its use as a natural fungicide. Proc. Natl. Acad. Sci. USA.

