# Supplementary material for: Cloning and Characterization of the Mycovirus MpChrV2 from Macrophomina phaseolina
Source: J Fungi (Basel). 2025 Sep 12;11(9):675. doi: 10.3390/jof11090675 (PMC12470682; doi:10.3390/jof11090675)
Supplement: Supplementary file 1 [file jof-11-00675-s001.zip › jof-3842812-supplementary.pdf]

Table S1. A list of primers used in this study.

Primer Name	Oligonucleotide sequence (5′- 3′)	Usage
RACE3RT	CGATCGATCATGATGCAATGCNNNNNN	For initial sequence cloning
RACE3	CGATCGATCATGATGCAATGC	For terminal sequence cloning
pC2	CCGAATTCCCGGGATCC	
pC3-T7loop	p-GGATCCCGGGAATTCGGTAA TACGACTCACTATATTTTATAGT GAGTCGTATTA-OH	
5-S-MPChrv2-1	TGGCGTGGTGGTGTAAGATG	
5-L-MPChrv2-1	CCAGATGCCATCTTCCCGAT	
3-S-MPChrv2-1	TCAAAGTGAAAAAGCGTCCGAT	
3-L-MPChrv2-1	AAAACACAGGGGTGGAGCC	
5-S-MPChrv2-2	GTCGTCTTCTTCGTTCATCAGC	
5-L-MPChrv2-2	CTGTATGTTACCAGGGCGAGGA	
3-S-MPChrv2-2	TGAGATAGGAGGAGTGCCGTTA	
3-L-MPChrv2-2	TGGTCCAGTAACACGGGTAAAG	
5-S-MPChrv2-3	AGAGCAGTGTTGCGATGGGT	
5-L-MPChrv2-3	GAACAACCAGCGGAACGAAT	
3-S-MPChrv2-3	AAGTCAAGTATGTCCTTCCGCA	
3-L-MPChrv2-3	GTATGTGCGATAGTGTGGAAGC	
5-S-MPChrv2-4	CGCCTCCACTTATTTGCACATT	
5-L-MPChrv2-4	TGTGCGTCTCACCCTCTAACTG	
3-S-MPChrv2-4	CGGTGAGGCTATGGTTGGACTA	
3-L-MPChrv2-4	GAAGGGCTCAGACAATGACAAC	
5-L-MPChrv2-3	GAACAACCAGCGGAACGAAT	
QC-MPChrv2-1-F	TGCAAAAAAGGAATAAAGGG	Full-length specific primers of the dsRNA1 segment of MpChrV2
QC-MPChrv2-1-R	TGAGTAATCAAGCTAGCTAGC	
QC-MPChrv2-2-F	GCAAAAAAGGAAAAAGGGG	Full-length specific primers of the dsRNA2 segment of MpChrV2
QC-MPChrv2-2-R	TGGTTAATCAAGCTAGCC	

QC-MPChrv2-3-F	TGCAAAAAAGGAATAAATGCCC	Full-length specific primers of the dsRNA3 segment of MpChrV2
QC-MPChrv2-3-R	TGAGTATCAAGCTAGCCAG	
QC-MPChrv2-4-F	TCTAGAGATTCGATCGATCATGATG	Full-length specific primers of the dsRNA4 segment of MpChrV2
QC-MPChrv2-4-R	TGAGTATCAAGCTAGCCAGCTAGC	
MpChrV2-1-F	TTTACACGACACTTCACG	Specific primers of the dsRNA1 segment of MpChrV2
MpChrV2-1-R	GTGAAAGCGGGAGTAAA	
MpChrV2-2-F	GCAGTACCGTTGACGAAGTT	Specific primers of the dsRNA2 segment of MpChrV2
MpChrV2-2-R	CCGAGACTCACCTCTCCTTT	
MpChrV2-3-F	GAAACAGCGGTATTGCCGTA	Specific primers of the dsRNA3 segment of MpChrV2
MpChrV2-3-R	CTTCCACTCAGTGCCTTGTC	
MpChrV2-4-F	TGCTGGGAAGGAAACT	Specific primers of the dsRNA4 segment of MpChrV2
MpChrV2-4-R	GTGACGCTGGATAAACTC	

Figure S1

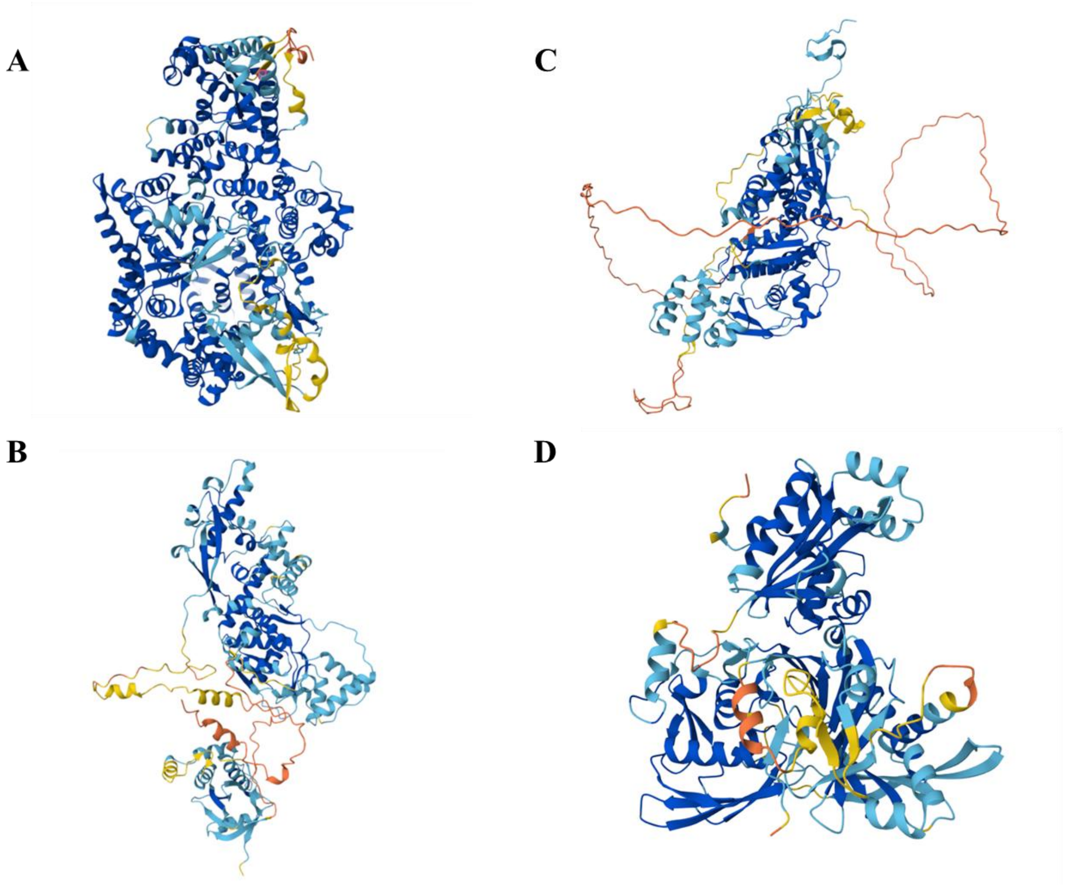

Figure S1. Predicted Tertiary Structure of MpChrV2. (A) Predicted tertiary structure of RdRp. (B) Predicted tertiary structure of CP. (C) Predicted tertiary structure of ORF3. (D) Predicted tertiary structure of ORF4.

Figure S2

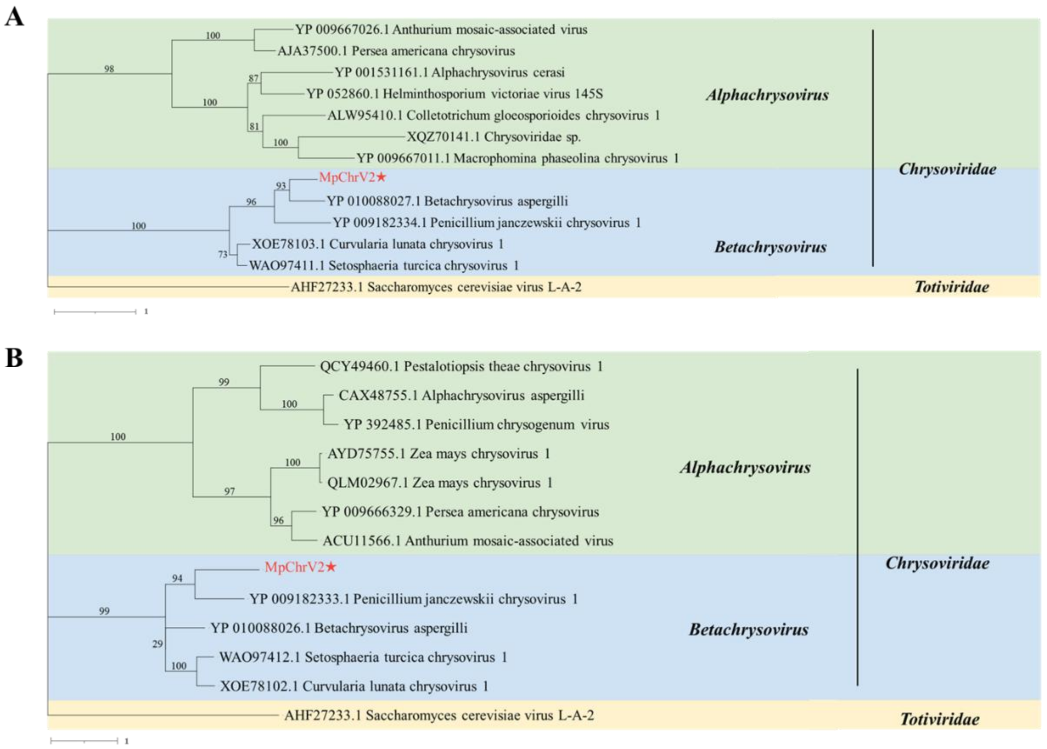

Figure S2. Phylogenetic analysis of MpChrV2 with other related viruses. (A) Phylogenetic analysis of the ORF3 of MpChrV2 and other mycoviruses. (B) Phylogenetic analysis of the ORF4 of MpChrV2 and other mycoviruses.
